# Somerby’s Concentrated Blow-pipe and Furnace

**Published:** 1845-04

**Authors:** 


### SOMERBY'S CONCENTRATED BLOWPIPE AND FURNACE.

We owe an apology to our friend, Dr. Somerby, for not having sooner noticed his ingenious and valuable invention, which, although designed chiefly for the dentist, is susceptible of being applied to so many purposes that it ought to be made known to all persons who have occasion to use a furnace or a blow-pipe. To the mineralogist and chemist, with slight modifications, this furnace is a machine of the greatest convenience, and to the dentist it strikes us as being invaluable. More soldering can be performed by it in an hour than can be done by the most skilful artist in six hours with the blast produced by his mouth. This is the experience of those who have tried it, and it is further stated on the same authority, that the largest piece of work put up by the dentist can be soldered in one minute, and in a manner superior to any other, the work when finished, presenting the appearance of having been cast in a mould. The furnace is so constructed that its outer surface is not affected by the heat, the varnish remaining uninjured by the most intense heat kept up within for hours together. On this account the instrument preserves its neat and beautiful appearance. There can be no doubt that it will be employed by every dentist in extensive practice, and that it will ultimately find its way into all the laboratories in the country. It is impossible to say how many uses it will serve, in how many processes and experiments it may be made to take part, and how much time and labor it is competent to save. We see that it is warmly commended in the American Journal of Dental Surgery, the highest authority on such subjects. Y.

